# Young maternal age is a risk factor for child undernutrition in Tamale Metropolis, Ghana

**DOI:** 10.1186/s13104-018-3980-7

**Published:** 2018-12-10

**Authors:** Anthony Wemakor, Humphrey Garti, Thomas Azongo, Helene Garti, Ambrose Atosona

**Affiliations:** 1 Department of Nutritional Sciences, School of Allied Health Sciences, University for Development Studies, P O Box TL 1883, Tamale, Ghana; 2 Department of Public Health, School of Allied Health Sciences, University for Development Studies, P O Box TL 1883, Tamale, Ghana

**Keywords:** Teenage mother, Stunting, Wasting, Underweight, Undernutrition, Tamale Metropolis, Ghana

## Abstract

**Objective:**

Malnutrition is a common cause of morbidity and mortality in children. The aim of this study was to compare the nutritional status of children under 5 years of teenage and adult mothers in Tamale Metropolis, Ghana. A case–control study involving 300 (150 cases, 150 controls) mother–child pairs was carried out. A questionnaire was used to collect data on socio-demographic characteristics of mothers and children and anthropometry was used to assess the nutritional status of children. Anthropometric z-scores derived based on WHO Child Growth Standards were used to determine stunting, wasting and underweight statuses of children. Logistic regression analysis was used to compare the nutritional status of children of teenage and adult mothers.

**Results:**

Children of teenage mothers, compared to those of adult mothers, were 8 times more likely to be stunted [Adjusted Odds Ratio (AOR) = 7.56; 95% confidence interval (CI) 4.20–13.63], 3 times more likely to be wasted (AOR = 2.90; 95% CI 1.04–8.04), and 13 times more likely to be underweight (AOR = 12.78; 95% CI 4.69–34.81) after adjusting for potential confounders. The risk of child malnutrition increases with young maternal age; interventions should be targeted at teenage mothers and their children to reduce the risk of malnutrition.

**Electronic supplementary material:**

The online version of this article (10.1186/s13104-018-3980-7) contains supplementary material, which is available to authorized users.

## Introduction

Adequate nutrition is one of the essential determinants of the health of children, and the right to adequate food is one of the fundamental human rights enshrined in many international agreements. Malnutrition is the most common nutritional disorder in developing countries [1, 2]. Globally, 22.9% of children under 5 years are stunted and 7.7% are wasted [3]. In Northern Region of Ghana, 33.1%, 6.3% and 20.0% of children under five are stunted, wasted and underweight respectively [4]. Malnutrition affects physical growth, morbidity, mortality, cognitive development, reproduction and physical capacity [2, 5]. About half of all deaths in children under five (about 3 million deaths) worldwide are caused by malnutrition [6].

About 11% of all births worldwide are to girls aged 15–19 years old and the vast majority of these (95%) occur in low and middle-income countries where child marriage is widespread [7]. In Ghana, 11.3% of females aged 15–19 years have had a birth, while 2.9% were pregnant at the time of interview [4]. Young maternal age at childbirth (< 20 years) is associated with increased risk of intrauterine growth restriction [8], low birth weight, preterm birth [9], infant mortality [10, 11] and poor child growth [12]. For example, in low and middle-income countries, babies born to mothers under 20 years of age have a 50% higher risk of being stillborn or dying in the first few weeks versus those born to mothers aged 20–29 years [7]. It is suggested that these associations result from interactions of biological, behavioural, and social factors. The nutritional needs of pregnant teenagers may compete with those of the developing foetus [13, 14] because they are still growing resulting in increased nutritional demands compared to adult pregnant women. Teenage mothers may breastfeed for a shorter duration than older mothers [15], be behaviourally immature and therefore less sensitive to the needs of their infants, and may easily get annoyed and use less emotionally positive communication compared to adult mothers [10]. In addition, younger mothers tend to have less education, and to be of lower socio-economic status and so have a higher chance of experiencing psychological stress resulting from limited resources [10] and parenting.

Despite the high prevalence of teenage childbearing in Ghana, data are scanty on the effects of low maternal age on growth indicators of Ghanaian children. The aim of this study was to assess the effect of low maternal age on child growth indicators by comparing the nutritional status of children under five of teenage and adult mothers in Tamale Metropolis, Ghana.

## Main text

### Methods

#### Study design, study population and sampling

A case–control study involving 300 children aged 6–59 months born to teenage (n = 150) and adult (n = 150) mothers in Tamale Metropolis, Ghana, was carried out from April to June, 2017. Teenage mothers (aged 15–19 years) and their children constituted cases while adult mothers (aged ≥ 20 years) and their children constituted controls. Subjects were recruited from four communities selected as a result of reports of high prevalence of teenage childbearing in the Metropolis: Kakpayili, Bulpiela, Kalariga and Kunyevilla.

The sample size of 300 (150 each for cases and controls) children was calculated using the formula of difference in proportions [16], 80% power, 95% confidence level, prevalence of stunting in control children, 33.1% [4] and determination of at least an odds ratio of 2. Simple random sampling was used to recruit both cases and controls.

#### Data collection

Pretested semi-structured questionnaire was used to collect data on socio-demographic characteristics of mothers and children and anthropometry was conducted on children. The weight of children was taken with a digital weighing scale with a least count of 0.1 kg and height was measured using an infantometer to the nearest 0.1 cm. Children who were 24 months and above were measured in the standing position and those who were less than 24 months were measured lying down. Anthropometric data were used to generate weight-for-height Z-score (WHZ), height-for-age Z-score (HAZ), and weight-for-age Z-score (WAZ) using WHO Anthro. HAZ, WHZ and WAZ < − 2 standard deviations from the median of the WHO Growth Standards were defined as stunting, wasting, and underweight respectively.

#### Statistical analysis

Data were analyzed using Stata IC (Version 12.1) and WHO Anthro. Means and standard deviations were calculated for continuous variables and frequencies and percentages for categorical variables. Chi-square test was employed in the bivariate analyses and variables that were significant in these analyses were considered as potential confounders in multivariate logistic regression analysis. Declaration of significance was done at p < 0.05.

#### Ethical considerations

Ethical clearance for this study was granted by the Joint Ethical Review Committee of the School of Medicine and Health Sciences and School of Allied Health Sciences, University for Development Studies (Protocol Number 02-2017). Written informed consent was sought from all study participants, and from the parents of 4 girls who were aged 15 years. All participants were assured of confidentiality of collected data.

### Results

#### Socio-demographic characteristics

The mean age of the mothers was 23.6 years and most (35.7%) were in the age group 18–19 years (Table 1). The majority of the mothers belonged to Dagomba ethnic group (76.3%), and had the fathers of children being household heads (77.7%), but about half of the household heads (51.3%) earned 500.00–999.00 Ghana Cedis (equivalent to approximately 100.00–199.00 United States Dollars at the time of going to press) monthly. The children were roughly evenly distributed by age and sex but the majority (72.0%) were born in health facilities.

**Table 1:** Socio-demographic characteristics of study subjects

Characteristic	Frequency (n = 300)	Percent
Age group of mother (years)		
15–17	43	14.3
18–19	107	35.7
20–24	33	11.0
25–29	55	18.3
30–34	27	9.0
35 +	35	11.7
Ethnicity of mother		
Dagomba	229	76.3
Gonja	47	15.7
Asante	9	3.0
Frafra	9	3.0
Others	6	2.0
Household head		
Mother	15	5.0
Father	233	77.7
Grand Parent	42	14.0
Others	10	3.3
Household head’s employment		
Unemployed	13	4.3
Farmer	53	17.7
Trader	89	29.7
Teacher	54	18.0
Nurse	8	2.7
Others	83	27.7
Household head’s monthly income (GHS)		
< 500.00	101	33.7
500.00–999.00	154	51.3
1000.00–1300.00	36	12.0
> 1300.00	9	3.0
Age group of child (months)		
6–11	107	35.7
12–23	99	33.0
24 +	94	31.3
Sex of child		
Male	147	49.0
Female	153	51.0
Birth place of child		
Health Facility	216	72.0
Traditional Birth Attendant	76	25.3
Home	8	2.7

#### Nutritional status of children

The average weight, height and anthropometric z-scores of children of teenage mothers were lower than those of children of adult mothers (Table 2). Overall, 39.0%, 8.0% and 16.3% of the children were stunted, wasted and underweight respectively. However, consistently, the prevalence of undernutrition was higher in children of adolescent mothers compared to children of adult mothers i.e., 59.3% versus 16.7% for stunting, 12.0% versus 4.0% for wasting, 29.3% versus 3.3% for underweight (Table 2).

**Table 2:** Mean anthropometric z-scores and prevalence of stunting, wasting and underweight, by mothers’ age group

Characteristic	All mothers (n = 300)	Teenage mothers (n = 150)	Adult mothers (n = 150)
Anthropometrics			
Weight (kg)	9.84 ± 2.59	8.44 ± 1.71	11.24 ± 2.56
Height (cm)	77.39 ± 11.84	71.39 ± 8.81	83.39 ± 11.46
Anthropometric z-scores			
Height-for-age (Mean ± SD)	− 1.62 ± 2.05	− 2.18 ± 2.28	− 1.07 ± 1.61
Weight-for-height (Mean ± SD)	− 0.08 ± 1.54	− 0.07 ± 1.24	− 0.08 ± 1.40
Weight-for-age (Mean ± SD)	− 1.34 ± 1.15	− 0.63 ± 0.82	− 0.99 ± 1.06
Nutritional status			
Stunting			
No (%)	185 (61.0)	61 (40.7)	125 (83.3)
Yes (%)	115 (39.0)	89 (59.3)	25 (16.7)
Wasting			
No (%)	276 (92.0)	132 (88.0)	144 (96.0)
Yes (%)	24 (8.0)	18 (12.0)	6 (4.0)
Underweight			
No (%)	251 (83.7)	106 (70.7)	145 (96.7)
Yes (%)	49 (16.3)	44 (29.3)	5 (3.3)

#### Association between maternal age and child nutritional status

In order to identify potential confounders to control for in the assessment of the effect of maternal age on child growth indicators, we compared the socio-demographic and economic characteristics for the two categories of mothers. This revealed statistically significant differences for type of household head (p < 0.001), occupation of household head (p = 0.025), household head’s monthly income (p = 0.001), and child’s age (p < 0.001) (Additional file 1). We therefore controlled for child’s age, type of household head and household head’s occupation in the adjusted logistic regression model. Children of teenage mothers, compared to those of adult mothers, were about 8 times more likely to be stunted [Adjusted Odds Ratio (AOR) = 7.56; 95% confidence interval (CI) 4.20–13.63)], about 3 times more likely to be wasted (AOR = 2.90; 95% CI 1.04–8.04), and about 13 times more likely to be underweight (AOR = 12.78; 95% CI 4.69–34.81) after adjustment for potential confounders (Table 3).

**Table 3:** Multivariate analysis comparing the nutritional status of children of teenage and adult mothers

Characteristics	Unadjusted Model		Adjusted Model^a^	
	Crude Odds Ratio (95% confidence interval)	p-value	Adjusted Odds Ratio (95% confidence interval)	p-value
Stunting				
Children of adult mothers	Reference		Reference	
Children of teenage mothers aged 15–17 years	9.33 (4.36–19.96)	< 0.001	9.97 (4.20–23.65)	< 0.001
Children of teenage mothers aged 18–19 years	6.89 (3.87–12.25)	< 0.001	7.08 (3.85–13.00)	< 0.001
All children of teenage mothers	7.50 (4.37–12.86)	< 0.001	7.56 (4.20–13.63)	< 0.001
Wasting				
Children of adult mothers	Reference		Reference	
Children of teenage mothers aged 15–17 years	3.16 (0.91–10.91)	0.069	2.48 (0.62–9.90)	0.198
Children of teenage mothers aged 18–19 years	3.32 (1.22–9.04)	0.019	3.01 (1.06–8.55)	0.038
All children of teenage mothers	3.27 (1.26–8.49)	0.015	2.90 (1.04–8.04)	0.041
Underweight				
Children of adult mothers	Reference		Reference	
Children of teenage mothers aged 15–17 years	17.19 (5.81–50.86)	< 0.001	19.80 (5.97–65.73)	< 0.001
Children of teenage mothers aged 18–19 years	10.28 (3.82–27.67)	< 0.001	11.49 (4.14–31.87)	< 0.001
All children of teenage mothers	12.04 (4.62–31.39)	< 0.001	12.78 (4.69–34.81)	< 0.001

^a^Model adjusted for child age, type of household head and household head’s occupation

### Discussion

We found increased risk of stunting, wasting underweight in children under 5 years of teenage mothers when compared to similar children of adult mothers in Tamale Metropolis, Ghana, suggesting an increased risk of child undernutrition with young maternal age.

In the present study, the overall prevalence rates of stunting and wasting in children under five: 39.0% and 8.0% respectively were higher compared to 33.1% and 6.3% respectively recorded for Northern Region in 2014 [4]. It was revealed that consistently, the prevalence of undernutrition for children of teenage mothers was higher than for children of adult mothers and the risk of undernutrition for these children was at least three times that of children of adult mothers. In agreement with the findings of our study, two studies observed higher prevalence of undernutrition among children belonging to teenagers compared to those of adult women in Accra and Bangladesh [10, 17].

The high prevalence of undernutrition overall recorded in our study population could be attributed to the high prevalence of undernutrition in children of young mothers. The main causes of undernutrition in children are inadequate dietary intake and infections so more children of young mothers might have been exposed to these. According to Mwaniki and Makokha [18], undernourished children have significantly inadequate intake of diversified foods and low rate of hand washing at critical times. Inadequate dietary intake and infections usually result from household food insecurity, inappropriate child care practices as well as poor health care [19].

Teenage mothers are less powered to guarantee children adequate dietary intake, access to safe water, and sanitary conditions given all the issues associated with teenage childbearing in Ghana. While their bodies may compete with developing foetuses being carried for nutrition, they may not be psychologically ready to nurse babies after delivery, or they may not have adequate financial resources to provide the needs of the babies owing to their poor socio-economic circumstances and that of their partners. Teenage mothers may not be accepted by their parents and may be forced to leave their care, their partners may also be teenagers with no stable source of income, they may face considerable stigma, or they may experience personal distress for getting pregnant prematurely and dropping out of school. As a result of these problems the amount and quality of care, nursing and nurturing they give to their children may be less compared to children of adult mothers. This is likely to affect the growth and development of their children resulting in malnutrition and other growth deficits.

### Conclusion

The present study revealed high prevalence of stunting, wasting and underweight among children under five of teenage mothers in Tamale Metropolis, Ghana. Children of teenage mothers compared to those of adult mothers were at least three times more likely to be undernourished. Efforts, including prevention of child marriages, should be intensified in order to prevent teenage childbearing. Teenage mothers and their children should be evaluated and specifically targeted with nutrition and health interventions to prevent undernutrition.

## Limitations

We used a cross-sectional study design which is not appropriate for studying cause-effect relationships and data were not available on the nutritional status of the mothers. This notwithstanding, we believe the present study sheds some light on the effect of young maternal age on child growth indicators in the Tamale Metropolis.

## Additional file

**Additional file 1.** Comparison of socio-demographic characteristics of teenage and adult mothers.

## Abbreviations

AOR: Adjusted Odds Ratio
CI: confidence interval
WHZ: weight-for-height
HAZ: height-for-age
WAZ: weight-for-age
WHO: World Health Organisation
