# Supplementary material for: Young maternal age is a risk factor for child undernutrition in Tamale Metropolis, Ghana
Source: BMC Res Notes. 2018 Dec 10;11:877. doi: 10.1186/s13104-018-3980-7 (PMC6288872; doi:10.1186/s13104-018-3980-7)
Supplement: Supplementary file 1 — Additional file 1. Comparison of socio-demographic characteristics of teenage and adult mothers. [file 13104_2018_3980_MOESM1_ESM.docx]

**Additional file 1: Comparison of socio-demographic characteristics of teenage and adult mothers**

| **Characteristic** |  | **Adult Mother** | **Teenage Mother** | **Test statistics** |
| --- | --- | --- | --- | --- |
|  | **N** | **Frequency (%)** | **Frequency (%)** |  |
| **Ethnicity** |  |  |  | X^2^=0.026; p=0.987 |
| Dagomba | 229 | 115 (50.2) | 114 (49.8) |  |
| Gonja | 47 | 23 (48.9) | 24 (51.1) |  |
| Others | 24 | 12 (50.0) | 12 (50.0) |  |
| **Household head** |  |  |  | X^2^=16.162; p<0.001 |
| Father | 233 | 131 (56.2) | 102 (43.8) |  |
| Others | 67 | 19 (28.4) | 48 (71.6) |  |
| **Occupation of household head** |  |  |  | X^2^=9.308; p=0.025 |
| Farmer | 53 | 27 (50.9) | 26 (49.1) |  |
| Trader | 89 | 51 (57.3) | 38 (42.7) |  |
| Teacher | 54 | 32 (59.3) | 22 (40.7) |  |
| Others | 104 | 40 (38.5) | 64 (61.5) |  |
| **Household head’s monthly income (GHS)** |  |  |  | X^2^=13.026; p=0.001 |
| <500.00 | 101 | 38 (37.6) | 63 (62.4) |  |
| 500.00-1000.00 | 154 | 81 (52.6) | 73 (47.4) |  |
| 1000.00+ | 45 | 31 (68.9) | 14 (31.1) |  |
| **Child age group (months)** |  |  |  | X^2^=48.186; p<0.001 |
| 6-11 | 107 | 31 (29.0) | 76 (71.0) |  |
| 12-23 | 99 | 46 (46.5) | 53 (53.5) |  |
| 24+ | 94 | 73 (77.7) | 21 (22.3) |  |
| **Sex of child** |  |  |  | X^2^=1.614; p=0.204 |
| Male | 147 | 79 (53.7) | 68 (46.3) |  |
| Female | 153 | 71 (46.4) | 82 (53.6) |  |
| **Birth place of child** |  |  |  | X^2^=0.265; p=0.607 |
| Health facility | 216 | 106 (49.1) | 110 (50.9) |  |
| Others | 84 | 44 (52.4) | 40 (47.6) |  |
